# Misinformation in Italian Online Mental Health Communities During the COVID-19 Pandemic: Protocol for a Content Analysis Study

**DOI:** 10.2196/35347

**Published:** 2022-05-20

**Authors:** Nicole Bizzotto, Susanna Morlino, Peter Johannes Schulz

**Affiliations:** 1 Faculty of Communication, Culture and Society Università della Svizzera italiana Lugano Switzerland; 2 Department of Communication and Media Ewha Womans University Seoul Republic of Korea

**Keywords:** online communities, social media, mental health, misinformation, COVID-19, empowerment, content analysis

## Abstract

**Background:**

Social media platforms are widely used by people suffering from mental illnesses to cope with their conditions. One modality of coping with these conditions is navigating online communities where people can receive emotional support and informational advice. Benefits have been documented in terms of impact on health outcomes. However, the pitfalls are still unknown, as not all content is necessarily helpful or correct. Furthermore, the advent of the COVID-19 pandemic and related problems, such as worsening mental health symptoms, the dissemination of conspiracy narratives, and medical distrust, may have impacted these online communities. The situation in Italy is of particular interest, being the first Western country to experience a nationwide lockdown. Particularly during this challenging time, the beneficial role of community moderators with professional mental health expertise needs to be investigated in terms of uncovering misleading information and regulating communities.

**Objective:**

The aim of the proposed study is to investigate the potentially harmful content found in online communities for mental health symptoms in the Italian language. Besides descriptive information about the content that posts and comments address, this study aims to analyze the content from two viewpoints. The first one compares expert-led and peer-led communities, focusing on differences in misinformation. The second one unravels the impact of the COVID-19 pandemic, not by merely investigating differences in topics but also by investigating the needs expressed by community members.

**Methods:**

A codebook for the content analysis of Facebook communities has been developed, and a content analysis will be conducted on bundles of posts. Among 14 Facebook groups that were interested in participating in this study, two groups were selected for analysis: one was being moderated by a health professional (n=12,058 members) and one was led by peers (n=5598 members). Utterances from 3 consecutive calendar years will be studied by comparing the months from before the pandemic, the months during the height of the pandemic, and the months during the postpandemic phase (2019-2021). This method permits the identification of different types of misinformation and the context in which they emerge. Ethical approval was obtained by the Università della Svizzera italiana ethics committee.

**Results:**

The usability of the codebook was demonstrated with a pretest. Subsequently, 144 threads (1534 utterances) were coded by the two coders. Intercoder reliability was calculated on 293 units (19.10% of the total sample; Krippendorff α=.94, range .72-1). Aside from a few analyses comparing bundles, individual utterances will constitute the unit of analysis in most cases.

**Conclusions:**

This content analysis will identify deleterious content found in online mental health support groups, the potential role of moderators in uncovering misleading information, and the impact of COVID-19 on the content.

**International Registered Report Identifier (IRRID):**

PRR1-10.2196/35347

## Introduction

### Online Communities for Mental Health Symptoms

In an online community, members can post their thoughts regarding a health condition or personal problems for other members to read and comment on. A support group communication episode is structured hierarchically, beginning with a person’s need for information or help (ie, locutor), to which any other member of the group may respond. Regarding the term *comment*, we also say post, utterance, or locution [1]. The reactions to a comment, if there are a fair number, would look like a branch of a tree split into ever more twigs. We refer to the whole branch as a bundle or a thread.

Some questions arising from this are evident: What are the influencing factors in the development of mental conditions? How do posts indicate changes in prognoses? But there are also other questions: Do posters stray? That is, do they prioritize other illnesses above or below their mental condition?

For instance, one of the key results in Cavazos-Rehg et al’s [2] study of Twitter was that depression and related factors were the subjects that drew the most attention by a large margin.

### Declarative and Procedural Knowledge

An origin or first post typically poses a question addressed to somebody’s declarative or procedural knowledge. Briefly speaking, declarative knowledge is a type of knowledge that can be verbalized and, on that basis, taught. Procedural knowledge is a person’s experience of how something is working [3]. Another typical demand in a first post would be for emotional support [4]. Griffiths et al [5] proclaimed to have captured the process rather than the end state of seeking support. Successful online support groups were also written about in scholarly journals, contributing to the impression that mental health support groups are an asset to a health care system [6,7].

### State of Health

In online communities for mental health symptoms (OCMHs), posts to groups and related comments have been deemed to sometimes have beneficial health consequences. Facebook-based social support, for example, was found to affect general health, mental illness, and well-being [4,8,9].

Regarding online communities, Nimrod [9] stated the following:

Findings indicated that online depression communities serve as a sphere for knowledge exchange, sharing the experience of living with depression, and getting inspiration for coping. Involvement in these communities seems to inspire and empower participants by enhancing a better understanding of their condition and encouraging them to ﬁght depression. Therefore, it is suggested that the communities can complement formal care.

Employing qualitative methods, Takahashi et al [10] found both better and worse states among the members of depression help groups. The authors especially highlighted the possibility of a downward spiral of a worsening state among groups.

### Correctness of Information

Deceptive or erroneous, rather than soothing or enlightening, information is also feared [11]. OCMHs may contain messages that contradict medical advice or reinforce unhealthy behaviors, such as those seen in Gavin and colleagues’ study [12], where anorexia and the misuse of diabetes medication to lower body weight were advocated on a webpage. The presence of misinformation was previously demonstrated in the context of antidepressant use on Instagram [13].

In a study of 6.5 million interactions generated by 500 posts, phatic posts constituted the strongest predictor of interactions, followed by posts with a positive emotional valence. Half of the posts were about social relations, and more than one-quarter (28%) consisted of health misinformation [14]. The article by Beck et al [15] reported on a content analysis that revealed emotional support to be the most frequently sought type of support and, at the same time, a majority of messages related to task rather than relation. Rueger and colleagues [16] reported that advice is more often followed when advice seekers perceive more similarities between themselves and advice givers (see Sillence [17]). The classical term for this is selective perception. If the perception of similarities between advice seekers and givers exerted some influence on advice acceptance, an adaptation by seekers regarding the type of internet service they chose was not observed [18].

A controversial subject is the correctness of internet content. However, recent articles posit the internet’s capacity to work against misinformation [19]. It is, however, unlikely that everyone who comes into contact with the misinformation will also be made aware of the correction. Different tensions may arise when the recognition of the increasing role of social media in health information consumption leads to the choice of traditional (ie, nonsocial) media for research [20].

The consequences of taking part in online communities can be differentiated into communicative and medical. Communicative consequences include ease of access to knowledge, enabling contact, or helping to find other people in a similar situation. These consequences are often considered the primary effects, as in a systematic review study by Moorhead et al [21]. The research methods in this area cross over into language use and mood analysis [22,23]. Medical consequences are the health effects, which are considered the secondary effects. One of the most engaging content analyses of online depression communities [9] summarized the influences of participation in terms of ameliorating the understanding of participants’ conditions and encouraging them to fight depression.

### Professional Moderation as an Uncovering Strategy

OCMHs can be moderated by health professionals; for instance, a health professional could prevent detrimental activity by establishing behavioral- and content-related rules and could moderate threads (eg, there could be restrictions on the mention of drugs and related matters). Moreover, they could censure or remove individuals from the OCMH [5]. Health professionals are defined in this study as people with a documented academic or professional background in the health field, but not restricted to mental health. These include psychiatrists or psychologists as well as nurses and general practitioners.

Very few studies have been conducted concerning the role of moderators and administrators (admins) in countering misinformation. Recent studies on moderation have shown that this type of intervention could improve the quality of online discourse (eg, Wadden et al [24]). However, these studies did not specifically investigate the impact of misinformation and its uncovering.

### Online Communities in the Midst of a Pandemic

Italy was the first Western country to experience a nationwide lockdown and this changed health priorities, with public health authorities advising the public to limit the use of health services [25]. Psychiatric emergency department admissions and referrals, in fact, diminished [26,27]. According to recent literature, this has led to the transition of many aspects of pre–COVID-19 life to a web-based format [28].

Although many studies focused on addressing the impact of social media during the pandemic (eg, the impact on panic [29] or on educating the public on prevention measures [30]), the reverse has not yet been investigated. A few studies have examined the characteristics of informational support or public sentiment related to COVID-19 (eg, Boon-Itt and Skunkan [31]), but a broader view on the impact on mental health conversations is still lacking. To the best of our knowledge, no study has been conducted to investigate whether people with mental health disorders turned more often to OCMHs or whether they gained or lost trust in the official health care system and encountered a higher amount of erroneous information.

An interesting question that arises is whether social media subjects changed as a response to the pandemic situation and whether the management of people’s mental conditions adjusted to the challenging situation.

Only one study investigated whether social media use increased after the onset of the COVID-19 pandemic; however, the community that was investigated did not specifically include people suffering from mental health disorders [32].

### Objectives and State of Knowledge

In this paper, we seek to describe how posts to OCMHs and their comments can form deceptive or erroneous information in terms of false declarative or procedural knowledge, wrong judgments, and motivations for detrimental decisions.

Furthermore, an exploratory research question asks whether misleading content would be distributed unequally at the different stages of illness trajectory. It is hypothesized that advice seeking or giving related to treatment, versus diagnosis or symptoms, will have the highest prevalence of misinformation.

Our study aims to do the following:

1. Add information about the influence of professional moderation on mental health communities to the knowledge base.
2. Demonstrate whether and how communication in mental health communities changes as a result of the COVID-19 pandemic.
3. Analyze the first two study aims for their role in misinformation and its detection.
4. Attempt to compare declarative and procedural knowledge demands.

This could be read as the foundation of a study that will test a complex causal model with demanding contemporary statistics, such as structural equation modeling. Instead, the method that will be used is a quantitative longitudinal content analysis [33]; 3 years of posts on a Facebook health community page will be analyzed, including some background and interpretive links.

## Methods

### Aspects of Content Analysis

Content analysis is a method to identify, record, and aggregate predefined elements of communication content [33,34]. The method assumes that the vast biological, educational, and social differences among the coders will not lead to a completely different understanding of similar text or other material. Selective perception has to be downgraded to the degree that different coders with different experience are able to understand and record communication content reliably. This is achieved by coder training and a collection of rules to understand and record the content; the rules are referred to as the codebook (Multimedia Appendix 1). The study will use utterances from Facebook OCMHs as the sampling frame. Facebook was chosen for several reasons: it is the largest social media company, it has a large presence of support groups, and it allows for easy retrieval and monitoring of member discussions, together with the number of threads present, compared to other types of OCMHs, such as blogs and forums.

### Ethics Approval

The guidelines outlined in previous social media research informed this study’s procedures [35]. Permission to conduct research was requested from admins or moderators of private and public Facebook groups. A group privacy setting of “private” means that while anyone can see the group, approval by an admin or another member is required to join; the content on a group’s wall is only visible to members [36]. In public groups, any Facebook user can join the group (ie, no admin permission is required).

Furthermore, regarding data that will be reported in scientific publications, we will remove any real names or other personal information, as well as usernames, that could potentially result in a breach of anonymity and privacy that would reveal any information that could be attributed to a single individual (eg, photographs and locations) [37]. In addition, researchers abstained from any communication or interaction with the individuals in the groups. Ethics approval was obtained by the ethics committee of the Università della Svizzera italiana on April 19, 2021 (CE 2021-4).

### Codebook Development

The first draft of the codebook was developed based on the categories identified in previously published works, for example, with respect to the distinction between information seeking and giving versus emotional support (eg, Greene et al [38] and Lerman et al [39]). However, in the later stages of codebook development, an inductive approach was applied as potential themes arose from the reading of data [40].

The second step in developing these coding schemes involved applying the codebook to a sample of Facebook threads in online support groups. This was done in an iterative process. This coding often resulted in the identification of further coding criteria linked to the variables of interest.

### Coder Training

Usually, content analysis coders do not have to have special skills. In this case, however, coders were required to have clinical experience and familiarity with mental conditions, and they were selected accordingly. Two coders were hired; both of them are psychologists with a background in health communication.

The two coders familiarized themselves with the coding procedure, the variables of interest, and the theoretical constructs underlying the codebook (eg, the concept of misinformation and the distinction between procedural and declarative knowledge). The coders were then assigned a number of threads (ie, consecutive posts) to code independently and to find shortcomings in the coding scheme. Codings were discussed among the coders and with the project management team. The iterative testing took place from November 2021 to January 2022.

When disagreement between coders and the management team had dropped to acceptable levels, the coders coded another 60 utterances for a reliability check, calculating a preliminary Krippendorff α. Coding problems and disagreements, as well as shortcomings in the codebook, were discussed, and the instrument was revised once more. In the next step, the two coders coded an additional 11 threads (61 utterances) and calculated the Krippendorff α for each variable coded (see the Results section).

### Sampling

#### Selection of OCMHs

Following a procedure suggested by Birnbaum et al [41], the project manager, in November 2021, searched for Italian-language OCMHs in the then-available versions that were present on Facebook. We chose the Italian language because most people posting in Italian would likely live in places influenced by Italian culture. If we had chosen English, a poster could live virtually anywhere and be subject to all kinds of cultural influences.

The ensuing recruitment process was visible to any member of the online groups found during the search in November 2021. The project manager entered a personal profile that was accessible to other group members. Using the personal profile, she requested to join the group, if private, and wrote a post on the group wall asking to be contacted by an admin or a group moderator. When contacted, she sent the same message to all the group moderators, which contained a brief presentation of the research team, the study aim, and the study methodology. When requests were made for additional information, she called the admins on the phone. In the event of no response, she wrote on the group wall twice and sent up to three reminders (every 2 or 3 days) to all of the group’s admins.

The aim was to select the most appropriate groups within the following categories: moderated by health professionals, moderated by peers, group size (ie, number of members), activity (ie, a group was considered active if posting at least 30 posts per month), and whether the group was already active since January 2019.

#### Selection of Material: Inclusion and Exclusion Criteria

##### Criteria for Origin Posts and Related Threads

The inclusion criteria for origin posts are follows: request information or advice in a decision situation, include calls for help regarding a health-related matter, or include calls for emotional help [42]. The reason for the restriction to these three types of origin posts was to concentrate as much as possible on real and severe issues. Posts will be also excluded when addressing relational communication among members in the group or when addressing group issues. Posts will be excluded when they appear to advertise professional services. All subsequent reactions to an excluded post will also be excluded. They will also be excluded when a moderator initiates the communication among members in the group or when the post is addressing group issues among members. The project management team will only pass along threads that meet the inclusion criteria and hold back those meeting the exclusion criteria.

##### Criteria for Posts

The project management team will only pass along posts that meet the inclusion criteria.

##### Criteria for Comments

Facebook has a nested comment feature that allows users to reply to individual comments on a post, with three maximum layers. In this way, subconversations among particular users can be collapsed and hidden into subthreads nested within a thread. Comments will be included only when posted at the first level; subthreads will not be considered as, according to the literature, they easily present parallel discussions [43]. No exclusion criteria are included for comments (ie, when an utterance is unrelated to the topic of discussion or deals with relational issues, this will be coded accordingly).

### Data Management and Coding Analysis

The project manager downloaded the threads and inserted them into a Microsoft Word file that already matched utterances (ie, posts and comments) with the sorting variable’s number (ie, the identifier that will be used later in the coding phase). In this step, emojis were replaced with keyboard symbols; see Multimedia Appendix 2 for examples of coding. A Microsoft Excel file was then created with the columns related to the formal variables (eg, date and the number of total comments in the post) already filled out.

Data were then coded by human coders, who entered their ciphers and letters into a data file in Microsoft Excel. Coders could correct obvious errors in sorting variables. Coders had help filling out the columns through the use of the IF function in Excel (eg, “NA” [not applicable] was automatically inserted for variables of advice giving when the utterance was coded as advice seeking).

After coding was complete, the data were transferred into the data matrix of a statistics program (SPSS); data cleaning was performed to make sure that there were no undefined codes in the file and that subgroup analyses were based on the correct filters and showed a reasonable number of posts or utterances. Generally speaking, content analysis can be based on counting different units. We will distinguish the individual utterances at a detailed level, which will make up the data matrix. 

Variables were coded on the locutor level and for all utterances, meaning that they do not form their own level. Every utterance has exactly one locutor. When a new utterance is added, a new coding line is opened, and the new utterance is coded in the new line under the given thread or within the same bundle. When the data file is cleaned, different entries for the same locutor are taken together.

### Variables

#### Overview

The codebook contains three types of variables: formal, locutor, and content variables; see Multimedia Appendix 1 for a complete list of the variables and coding details and Multimedia Appendix 3 for a summary.

#### Formal Variables

The bundle and sorting variables (V1 and V2) have the technical function of distinguishing comments from different threads and monitoring the related timeline of comments. V3 codes the number of total comments in the thread. V4 (ie, locution date) is coded based on the first utterance in the thread. To allow for comparisons between the times after the COVID-19 pandemic had hit and before, the study precisely records the date of publication. That allows for recoding of dates into any period one could wish for. V5 codes for reactions, such as likes or loves; this can be coded for all utterances (Multimedia Appendix 2).

#### Locutor Variables

The locutor variables include locutor ID (V6), to track interventions within and between threads, and locutor gender (V7). Locutor status (V8) was coded for every utterance, though the status will likely be the same in all utterances for one locutor within a bundle. However, change will not be ruled out, and one and the same locutor can have different statuses across bundles and within bundles. Furthermore, the medical-scientific qualification of the locutor (V9) will be coded if mentioned anywhere in the locution.

#### Content Variables

##### Overview

These variables were coded at the comment level. V10 has the purpose of distinguishing when the locutor is seeking or giving advice. A code of “seeking” represents the seeking of advice, information, decision help, or emotional help. Requesting one of the types of support often includes, implicitly or explicitly, the admission of one’s limited abilities or energy, of failure, or of weakness. The origin utterance should, as a rule, be coded as seeking, also due to the inclusion criteria. The categories include the following: declarative knowledge question (ie, know that), procedural knowledge question (ie, know how), asking for help in making a health decision, and asking for emotional support. Later, other members of the support group may join in making the same or other requests. A code of “giving” means that the locutor did, or tried to do, what was requested. First, the success or failure of the locutor is not of import to the coding. To code an utterance as a reaction, an explicit link must be there. In this variable, a value of 0, which represents that neither giving nor seeking was indicated, aims to facilitate coding for utterances that do not indicate advice seeking or giving (eg, problems within the Facebook group, greetings, or requests for further information).

There will be a filter here, with posts coded as 0 stopping the coding, those coded as seeking being directed to V11 (ie, motivation for a seeking locution), and those coded as giving being directed to V12 (ie, action through a giving locution). The categories are as follows: answer to a declarative knowledge question, answer to a procedural knowledge question, call to action, and providing emotional support.

In summary, we can connect the content variables that are especially important for this study as follows:

- Variables regarding the effects of the pandemic: V11 to V13
- Variables regarding the role of the professional: V16 and 17
- True and false variables: V14 to V19
- Declarative and procedural variables.

For V11, we code what the origin post or any other locutor actually wants, if seeking. This is done with four rather broad and abstract categories; this is the basis of a fundamental division we have drawn. The categories are as follows: an answer to a declarative knowledge question (ie, know that), an answer to a procedural knowledge question (ie, know how), asking for or giving help in making a health decision, and asking for or giving emotional support. For V12, we also code the answer of a responding group member in terms of declarative knowledge (ie, the locutor provides declarative knowledge to the recipient), procedural knowledge, call to action (ie, the locutor makes a referral to a health professional), and emotional support.

The type-of-illness variable (V13) is coded in the following way. The utterance is coded for the disease it relates to; up to three diseases can be coded. This can be identified more explicitly (ie, direct mention of the illness) or implicitly (ie, through drugs mentioned). The treatment options variable (V14) indicates which treatment options are communicated in an utterance. It is possible to code more than one treatment option; medication types will be identified through Multimedia Appendix 4*.* This variable contains 16 categories grouped as follows: hospitalization, psychotherapy, medication, surgical interventions, and alternative interventions. The treatment evaluation variable (V15, a-c) indicates how the locutor evaluates the treatment (a) together with the presence of the mention of (b) adverse effects and (c) treatment interruption. When a locutor suggests a treatment, this is coded as if the treatment is evaluated positively.

Furthermore, V16 (a-c) investigates (a) whether a health professional is mentioned, (b) the related sentiment *toward* the health professional, and (c) the doctor-patient relationship. Notably, two mental health professional types—psychologist and psychotherapist—will be coded in the same category (value of 1), because when pilot-testing the codebook, users in online support groups rarely distinguished correctly between the two.

Furthermore, the argument quality used by the advice giver is coded in V17. The categories are as follows: empirical knowledge, professional knowledge, secondhand professional knowledge, secondhand unprofessional knowledge, direct anecdotal evidence, or indirect anecdotal evidence.

Misinformation will be investigated in V18. Misinformation is defined as “cases in which people’s beliefs about factual matters are not supported by clear evidence and expert opinion” [44]. Misinformation will be categorized into content-related and context-related types. Content-related misinformation refers to situations in which the utterance does not present evidence in support of other claims that used poor-quality evidence or no clinical evidence to support them. On the other hand, context-related misinformation refers to when, irrespective of the quality of the information communicated, the information is not adequate for the context as (1) the locutor does not hold all the knowledge necessary to justify their comment or (2) the locutor does not have the status to be able to make certain inferences. Furthermore, a category specific to misinformation in advice seeking was added to classify when the locutor asks for help while basing the request on wrong assumptions (eg, “antidepressants don’t work for me, can you recommend a natural method?”). A category (ie, specifier) was added for utterances containing incorrect terminology, such as stigmatizing or inappropriate terms. Furthermore, misinformation correction will be also measured dichotomously. Misinformation correction will be considered not only as “the presentation of information designed to rebut an inaccurate claim or a misperception” [45], but also when a locutor simply expresses disagreement with a previous misinforming utterance.

**Figure 1:**
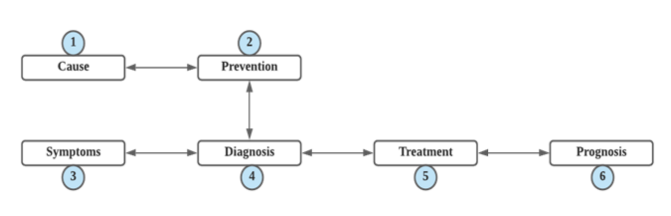
Illness trajectory. The boxes represent the cornerstones and the arrows represent the themes.

V19 is dedicated to the investigation of the illness trajectory and aims to identify at which stage the advice seeker needs informational or emotional help. This does not identify the stage at which the locutor is at the moment, but the stage at which the request can be collocated. Illness trajectories include the normal course of events and developments that span from either the patient or a physician noticing something is wrong up to successful or failed treatment. The trajectory standardizes what people think or say about illness and, thus, enables quantitative coding. It consists of six cornerstones: (1) causes and risk factors, (2) prevention, (3) symptoms of mental conditions, (4) diagnoses that support-seekers converse about, (5) treatment, and (6) prognosis. These six cornerstones describe and structure the illness trajectory. The pairwise combination of two cornerstones creates a theme, for instance, which symptoms indicate which diagnosis. In Figure 1, the boxes represent the cornerstones and the arrows represent the themes (ie, pairwise beliefs); some themes can be constituted by cornerstones that are not next to each other. We turn to the cornerstones first, followed by the themes.

##### Causes and Risk Factors Cornerstone

Causes necessarily come at the early stage of the trajectory. When the disease moves into a patient’s focus, the disease’s causes are no longer as interesting as they would have been, had a patient been aware of what condition they would develop. This appears in the trajectory as prevention.

##### Prevention Cornerstone

If causes are known or assumed, a rational decision can be made of what could be done; for instance, if it is known or believed that lack of exercise causes coronary disease, a patient would have to exercise more to protect themself from this condition.

##### Symptoms Cornerstone

Receiving information about newly noticed or, in fact, new symptoms is expected to be a frequent subject on support websites and a frequent motive for posting an origin locution. Attention to and worry about symptoms will be linkable to diagnosis. The corresponding locutions are statements regarding which symptoms point to which diseases. An example would be “Sudden loss of body weight without a change in diet or an uptake of exercise is often a sign of depression.”

##### Diagnosis Cornerstone

This cornerstone represents the diagnosis or condition that is discussed in the utterance, not a diagnosis that anyone in the support group might or might not have. Categories of diseases were taken from the Diagnostic and Statistical Manual of Mental Disorders, Fifth Edition (DSM-5). The structure of the conditions were also taken from the DSM-5, which enables coding of an imprecise use of language.

##### Treatment Cornerstone

Treatment decisions, in reality, often have several criteria to consider and weigh against one another. All of the utterance categories are, of course, situation specific. Therefore, the recommended treatment may not be specific and, in discussions, it may often be reduced to either psychotherapy or medication.

##### Prognosis Cornerstone

This cornerstone encompasses the prospect of recovery along with adverse effects and medication interruption. An adverse effect is an undesired harmful effect resulting from a medication or other intervention.

##### Themes

Themes were then identified as entities that are defined as pairs of cornerstones, such as “causes and risk factors in relationship with symptoms” or “symptoms in relationship with diagnosis.”

## Results

### Codebook Development

We first demonstrated the usability of the proposed systematic framework with 11 threads (61 utterances) coded independently by two coders. The KALPHA macro for SPSS for Windows (version 26; IBM Corp) [46] was used to calculate the Krippendorff α; the coders reached intercoder reliability (α=.89, range .73-1). Then, 1534 units of analysis (144 threads) from two Facebook groups were retrieved and analyzed by two coders. Intercoder reliability was calculated on 293 units (19.10% of the total sample; Krippendorff α=.94, range .72-1).

The α values were calculated for the following: locutor gender (α=1), locutor status (α=1), medical-scientific qualification of locutor (α=1), seeking versus giving advice (α=1), motivation for seeking advice (α=.93), action through giving locution (α=.95), type of illness (α=.95), treatment options (α=.98), treatment evaluation (α=.93), adverse effects of treatment (α=.93), treatment interruption (α=.94), health professional mentioned (α=.97), sentiment toward health professional (α=.72), doctor-patient relationship (α=.95), argument quality (α=.93), misinformation (α=.90), misinformation correction (α=.91), and illness trajectory and themes (α=.98).

### Content Analysis

In total, we have contacted 21 groups; five groups were public but were still asked for approval. Of these groups, 14 agreed to participate in the study. Among those who agreed to participate, according to the selection criteria, we chose two groups: one moderated by a health professional (n=12,058 members) and the other led by peers (n=5598). Information on the number of participants was retrieved in November 2021.

Analytic procedures will be determined in detail. In addition to descriptive statistics of the variables considered, the analysis will combine different units of analysis levels (eg, utterances in relationship to threads). The independent variables will be recoded from the locutor variables and the locutor role of seeking advice versus giving advice: put simply, the seeking of posters, their qualifications, and the illnesses by which they are afflicted. Interfering variables will be the seeking and giving dichotomy, the type of illness, the treatment mentioned, the sentiment toward the health professional, and the argument quality. Misinformation and misinformation correction will be the principal dependent variables.

The study will contribute to investigating potential detrimental effects, and possible mitigating factors, of OCMHs.

## Discussion

### Expected Findings

Findings will be explained by the concepts of health literacy and health empowerment. In the context of the two concepts, certain communication elements of health subjects are associated with erroneous utterances. Working with the two concepts presents the problem of a striking plurality of concepts as well as operationalization. We, therefore, will not link measures or data, but will look at the origin of erroneous health beliefs and detrimental decisions and will provide the link by way of interpretation.

Consequently, health literacy and empowerment were not coded anywhere in this content analysis. In spite of this, some understanding of the theories behind data collection might help us understand what is being measured and why. Therefore, a few more words on health literacy and empowerment are added here. Health literacy can be defined as the “capacity of individuals to obtain, process, and understand the basic information and services needed to make appropriate health decisions” [47]. Knowledge is not mentioned but is identified in the same writing as a moderator of health literacy. It is certainly a product of literacy and a facilitator of further knowledge acquisition [48]. This position of knowledge and health literacy gives us reason to consider the discussion, application, and evaluation of knowledge as major elements of erroneous consequences of health literacy.

Empowerment, in general, was defined as regaining mastery of one’s life [49]. That concise formulation can be specifically applied to health empowerment, which would be limited to matters of health, including prevention, care, disease, and treatment. Empowerment becomes harmful when thinking about and discussing competence in making medical decisions. Competence has the same meaning as in legal provisions or social norms: granting the *power* to make decisions. Competence in another sense refers to the *ability* to make decisions. That sense of the term belongs to health literacy rather than empowerment. Expressed in modal verbs, competence regarding legal and social norms that is related to empowerment is about what you *may* (ie, are allowed to) do; the other sense is related to health literacy and is about what you *can* (ie, are able to) do. Agreeing to nonprofessional decision-making (ie, supporting a patient in the belief that he does not need to seek medical consultation) belongs here. Keeping a patient away from a doctor when he or she should see one is the second element of deleterious consequences, this one resulting from an overestimation of empowerment. An example is the linkage between empowerment and using internet health services [50].

Aside from a few analyses that will compare distinct threads, individual utterances will constitute the unit of analysis in most cases [51]. More specifically, we expect misinformation to be more prevalent at specific stages of the illness trajectory, such as the treatment stage, and that professionally moderated OCMHs will present a lower number of misleading messages or negative comments toward health professionals and treatment options.

Furthermore, in addition to COVID-19 impacting the subjects of discussion, we expect the highest amount of misinformation to occur during the pandemic year 2020.

### Strengths and Limitations

To our knowledge, this is the first piece of research that will examine OCMHs using the methodology of longitudinal quantitative content analysis. The study will permit us to overcome the limitations of automatic text analysis. However, limitations should be taken into consideration, for instance, female and male genders cannot always be reliably inferred from names, and this categorization limits the variety of gender identities.

### Future Directions

We are not yet aware about the impact of OCMH messages on members’ intentions and behaviors in terms of professional help seeking or attitudes toward health professionals and treatment options. Future research may wish to explore this aspect.

## Appendix

Multimedia Appendix 1 Codebook.

Multimedia Appendix 2 Reactions and emojis.

Multimedia Appendix 3 Summary of variables.

Multimedia Appendix 4 Medication types.

## Abbreviations

admin: administrator
DSM-5: Diagnostic and Statistical Manual of Mental Disorders, Fifth Edition
OCMH: online community for mental health symptoms
V: variable (when used with a number)
