# Supplementary material for: Misinformation in Italian Online Mental Health Communities During the COVID-19 Pandemic: Protocol for a Content Analysis Study
Source: JMIR Res Protoc. 2022 May 20;11(5):e35347. doi: 10.2196/35347 (PMC9166639; doi:10.2196/35347)
Supplement: Multimedia Appendix 1 [file resprot_v11i5e35347_app1.docx]

Multimedia Appendix 1: Codebook

This is a Multimedia Appendix to a full manuscript published in the J Med Internet Res. For full copyright and citation information see <http://dx.doi.org/10.219/35347>

# Procedure

First of all, the thread should be entirely as information from the conversational thread structure can serve as contextual constraints to better capture the meaning of a single utterance [1].

Then, utterance should be coded in chronological order.

# Variables and Categories

## Formal variables

### V1 Bundle number

Should already be contained in the sorted material that coders receive from project management. Assign them consecutively.

### V2 Sorting variable

That is a device to reconstruct the position of an utterance in the chronological order.

### V3 Number of total comments in bundle

### V4 Locution date

Date the locution was shared (DD/MM/YY format). This is only coded for the serial utterance.

### V5 Reactions

Reactions are smiley-type drawings and can be: Like, Love, Care, Haha, Wow, Sad and Angry). Number and type of reactions are expressed in this way: 9L = 9 likes, 2W = 2 wows. In case of fragmented utterances, the number of likes will be represented by the number present in the utterance with the highest number of reactions [2].

## Locutor variables

These variables are coded for all utterances. When a new utterance comes, a new coding line is opened under the given thread or within the same bundle, and the new utterance is coded in the new line.

### V6 Locutor ID

Once the text body for analysis is secured (downloaded, printed) all the threads, locutor will be assigned a numerical code to be able to track his interventions (posts and comments) in different threads. This is made possible as Facebook adopt a real-name system so that names remain the same across threads [3]. The locutor name will be recoded across threads with the “Find [name] and Replace [ID]” function of MS Word.

### V7 Locutor gender

Gender is derived from nominal information: 1 = female, 2 = male**.** This is made possible as according to recent studies the vast majority of the Facebook users have account in real name and Facebook policies require users to have a Facebook profile with their legal name [4].

### V8 Locutor status

From all information available, code the role that corresponds best to what the locutor does and is. The list of the names of the moderators in the groups will be at hand during this step to identify moderators.

| 10 | Locutor is a patient |
| --- | --- |
| 20 | Locutor is a moderator |
| 31 | *Locutor is a caregiver, his/her role is:* Patient’s partner |
| 32 | *Locutor is a caregiver, his/her role is:* Patient’s parent |
| 33 | *Locutor is a caregiver, his/her role is:* Patient’s daughter /son |
| 34 | *Locutor is a caregiver, his/her role is:* Patient’s sister /brother |
| 35 | *Locutor is a caregiver, his/her role is:* Patient’s friend, neighbor, acquaintance |
| 36 | *Locutor is a caregiver, his/her role is:* Other relative (specify) |
| 40 | Ex patient |
| 50 | Locutor is a mental health professional |

E.g., to be coded 10 "Hello everyone, I have been suffering from depression since summer 2015"

E.g., to be coded 36 “Good evening to everyone and thanks for the space you give us. I write for my mom. The general practitioner has been prescribing five drops of Compendium for her anxiety problems since 2017. "

E.g., to be coded 33 “My boyfriend and I (we've been together for 8 years) just broke up. He says he has to keep up with his mum who is suffering from depression and his grandmother who has trouble moving. We wanted to move in together but in this situation, it is impossible we will never have a life of our own. My heart is in pieces, I feel terrible."

### V9 Medical-scientific qualification of locutor

We code the highest qualification of the locutor that is mentioned anywhere in the locution. This can be mentioned in the Facebook name (e.g. Doctor XY) or in the conversation.

| 0 | No information |
| --- | --- |
| 1 | Approbation as psychologist, psychiatrist, neurologist and comparable disciplines |
| 2 | Approbation in other medical fields |
| 3 | Other, non-medical but relevant qualification e.g. hairdresser |
| 4 | Alternative medicine practitioner e.g. acupuncturists, naturopath |

## Content variables

### V10 Seeking vs. giving

This is to code the major motivation or intention the locutor pursues. Seeking means the seeking of advice, information, decision help, emotional help. These categories are identical with the inclusion criteria. Requesting one of the types of support often includes, implicitly or explicitly, the admission of one’s limited abilities or energy, of failure. or of weakness.

The origin utterance should, as a rule, be coded as seeking also in line of the inclusion criteria. Later other members of the support group may join in asking the same or other requests.

Code “Giving” is chosen when the locutor reports what he or someone else did, tried to do, or intends to do with regard to the request. First, the success or failure of the locutor is not of import to the coding. The cases that are coded “Seeking” allude to a demand, a motivation. Cases classified as “Giving” suggest activity. To code an utterance as a reaction, an explicit link must be there.

| 0 | Neither giving or seeking is indicated (go on coding V15) |
| --- | --- |
| 1 | Seeking (go on coding *V11*: *Motivation for “seeking” locution, then skip V12 and go on with V13* ) |
| 2 | Giving (*skip V11 and go on with V12*: *Action through “giving” locution*) |

### V11 Motivation for “seeking” locution

| 1 | **Declarative knowledge question (know-that)**  The origin locutor presents him/herself as lacking knowledge in a concrete field and puts the question to the public (so to speak). Declarative knowledge is knowledge of the fact that something is there, something exists. It can be put into words rather easily. |
| --- | --- |
| 2 | **Procedural knowledge question (know-how)**  Procedural knowledge is the knowledge exercised in the accomplishment of a task. Request for concrete, direct, and specific suggestions, direction, or guidance about possible courses of action. It comprehends for example: - Explicit request of recommendation for a physician, specialist, hospital or another medical facility - What will happen during a clinical visit - How to manage medications |
| 3 | **Help in making a health decision**  Origin locutor demands help in making a mental health decision. The origin locutor presents him/herself as being unable to make the decision. Demanding decision assistance can be split into three questions: Can I make the decision myself? And if not, who will be deciding for me? And why should I trust that person or institution? A knowledge locution may well be instigated by a decision problem. We code either answering a knowledge question or helping a decision, depending on how the origin locutor frames his/her problem. |
| 4 | **Emotional support seeking** Presence of venting and emotional expression. Empathic story-sharing. Emotional and not informational support is required. In case of a locutor writing as origin utterance only what he/she is feeling without specific requests for help, this will be classified as emotional support seeking. Emotional support seeking will be coded also when users offer for a private chat. |

E.g., to be coded 1 “*Do any of you associate two antidepressants? What results do you have?*"

E.g., to be coded 2 "*How can I tell if I have fallen into depression again?*"

E.g., to be coded 3 “*As I said before, however, the reason for my visit was another, so I don't know whether to give up this treatment and go to the neurologist to remove my doubt about borderline disorder, or to start this treatment suggested by her. I'm a little afraid of drugs, I know it's stupid but taking two at the same time scares me a little. I know it's a personal decision and I'll definitely do my own thing, but what do you recommend? Thanks to those who will answer* "

E.g., to be coded 4 "*Help me*"

### V12 Action through “giving” locution

| 1 | **Declarative knowledge answer (know-that)**  Locutor provides declarative knowledge |
| --- | --- |
| 2 | **Procedural knowledge answer (know-how)**  Locutor provides procedural knowledge |
| 3 | **Call to action**  Call to action can be considered a specific type of procedural knowledge answer that occur when locutor refer the advice-seeker to a health professional |
| 4 | **Emotional support giving** Locutor provides emotional support |

E.g., to be coded 1 “*I don’t think that all antidepressant have the same active ingredient*"

E.g., to be coded 2 " *To book a doctor’s appointment you have to…*"

E.g., to be coded 3 “I *think the best option is to call your psychiatrist and ask him about the adverse effects*“

E.g., to be coded 4 “*I am sad and sorry to hear your story. I hope things get better*”

### V13 Type of Illness

The origin locutor’s request may be related to a specific disease or to several or all of them. That might be true of later utterances, too. There is no reason to assume that later utterances are on the same disease. Therefore, every utterance is coded for the disease it relates to as there may be more than one disease be spoken about in a single utterance. We allow there to code up to three diseases. Be certain that all ensuing variables that take up and relate to the three diseases are put in a column that fits. For example, if the type of illness is coded as a): depression, b) bipolar and c) personality disorders, then the treatment options in V14 must be coded: a) treatment of depression, b) of bipolar and 6) of personality disorders.

| 1 | Depressive and related disorders (Disruptive Mood Dysregulation Disorder, Major Depressive Disorder, Persistent Depressive Disorder or Dysthymia, Premenstrual Dysphoric Disorder) |
| --- | --- |
| 2 | Anxiety and related disorders (Separation Anxiety Disorder, Selective Mutism, Specific Phobia, Social Anxiety Disorder or Social Phobia, Panic Disorder, Agoraphobia, Generalized Anxiety Disorder) |
| 3 | Bipolar and related disorders (Bipolar I, Disorder Bipolar II, Disorder Cyclothymic Disorder) |
| 4 | Obsessive-Compulsive and related Disorders (Body Dysmorphic Disorder, Hoarding Disorder, Trichotillomania, Excoriation Disorder) |
| 5 | Substance-Related and Addictive Disorders (Alcohol, Caffeine, Cannabis, Tobacco) |
| 6 | Personality disorders (Paranoid, Schizoid, Schizotypal, Antisocial, Borderline, Histrionic, Narcissistic, Avoidant personality disorder) |
| 7 | Other mental illnesses (Feeding and Eating Disorders, Neurocognitive Disorders) |
| 8 | Unspecified mental illness |
| 9 | Physical illness |
| 10 | COVID-19 |
| 11 | Suicidal ideation |

E.g., to be coded 1,2 “I suffer from anxiety and depression"

E.g., to be coded 11 “*At night I have bad thoughts and I think I would like to disappear*”

### V14 Treatment options

This variable indicates which treatment options are communicated in an utterance. Importantly, when a locutor refers to a specific health professional, by speaking of a person (e.g., “the Psychologist” or “the Psychiatrist”) this will usually be considered a person, that is coded under *V18*. On the other hand, if locutor refers synecdochically to “the Psychologist” or “the Psychiatrist”, this will be coded, the utterance has to be coded in terms of the present variable of Treatment options (*V14*) rather than *V18*. When locutors mention “cure” in general, code as if they mentioned psychotherapy and medications.

We allow there to code up to three treatment options.

| 0 |  | **No treatment mentioned** |
| --- | --- | --- |
| 1 |  | **Psychotherapy in general** |
|  | 11 | Cognitive behavioural therapy |
|  | 12 | Psychoanalysis and psychodynamic therapies |
|  | 13 | Other types of psychotherapy |
| 2 |  | **General medications** *(See Multimedia Appendix 2)* |
|  | 21 | Drugs generally used to treat depression (antidepressants) |
|  | 22 | Drugs generally used to treat anxiety (anti-anxiety medications) |
|  | 23 | Drugs generally used to treat psychotic disorders |
|  | 24 | Mood stabilizers |
|  | 25 | Drugs to Treat Insomnia |
|  | 26 | Other types of medication |
| 3 |  | Alternative mental and mind-body interventions interventions (e.g., respiration, yoga, meditation, hypnosis, self-help) |
| 4 |  | Alternative physical interventions (e.g., hypericum, homeopathy, essential oils, herbal interventions, CBD oil nutritional supplements and aromatherapy, Bach flowers, acupuncture) |
| 5 |  | Surgical, as e.g., brain stimulation as used to treat Parkinson |
| 6 |  | Hospitalization |
| 7 |  | COVID-19 Vaccine |

E.g., to be coded 11 “*I have not been well for a long time, I have changed therapists and I am doing Cognitive Behavioral Therapy / Schema Therapy*”

### V15 a-c Treatment evaluation

This variable indicates evaluation of the treatment in Columns a) b) or c) of the previous two variables

1. Sentiment towards treatment

| NA | No treatment mentioned |
| --- | --- |
| 1 | Treatment – neutral |
| 2 | Treatment judged positively |
| 3 | Treatment judged negatively |
| 4 | Mixed (there are both positive and negative nuances) |

E.g., to be coded 3 “*They gave me medicine but it makes me even worse”*

1. *Treatment adverse-effects*

| NA | No treatment mentioned |
| --- | --- |
| 0 | No adverse effects mentioned |
| 1 | There is mention of adverse effects. *E.g., A locutor asks what are the side effects of antidepressants* |

E.g., to be coded 3 “*Antidepressants just make me dizzy and nauseous”*

1. *Treatment interruption*

Can have occurred because of adverse-effects or ineffective treatment. This can be mentioned in terms of past or present behaviour or of intentionality. Moreover, we do not include in thi category scheduled treatment interruptions (intermittent therapy) or interruption because of healing.

| NA | No treatment mentioned |
| --- | --- |
| 0 | No treatment interruption mentioned |
| 1 | There is mention of treatment interruption. *E.g., A locutor tells that s/he interrupted treatment because it did not work and s/he suffered from many adverse-effects such as dizziness and weight gain* |

E.g., to be coded 1: “*Yesterday I stopped taking antidepressants without asking my psychiatrist: I was too sick. Do you think it could be a problem?”*

### *V16 a,b,c Health professional mentioned and related sentiment*

We allow there to code up to two health professionals.

1. *Health professional mentioned*

| 0 | No |
| --- | --- |
| 1 | Psychologist or psychotherapist |
| 2 | Psychiatrist |
| 3 | Unspecified mental health professional |
| 4 | Other health professional (clearly not mental health specialist) |
| 5 | Non-professional source (e.g., a spiritual healer, shaman) |

1. *Sentiment towards health professional*

Sentiment can be identified through locutors’ use of specific verbs, adverbs or adjectives that correspond to a polarity classification.

| NA | Health professional not mentioned |
| --- | --- |
| 0 | Do not express |
| 1 | Health professional judged positively |
| 2 | Health professional judged negatively |
| 3 | Mixed opinion on health professional |

E.g., to be coded 2 *“I have been suffering for 30 years. They sent me to the psychologists but I did not solve anything on the contrary it got worse”*

1. *Doctor-patient relationship*

Locutor discusses about his relationship with the doctor.

| NA | Doctor-patient relationship not mentioned |
| --- | --- |
| 0 | Do not express |
| 1 | Doctor-patient relationship judged positively |
| 2 | Doctor-patient relationship judged negatively |
| 3 | Mixed opinion on Doctor-patient relationship |

E.g., to be coded 2: “*I do not trust him. I think he takes advantage of my money*”

### V17 Argument quality

It is coded which type of evidence an utterance contains to support the advice given. The variable is coded for all utterances coded 2 or 3 in Seeking vs. giving (V10).

| NA | Not giving-behaviour |
| --- | --- |
| 0 | No explicit reference to the argument quality |
| 1 | **Direct anecdotal evidence** Comments that express knowledge gained from own personal experience and own participation in a particular phenomenon (e.g., knowledge about how to deal with depression gained by coping with one’s own depression). An utterance to be coded in this category has to explicitly mention that knowledge on the topic was gain through personal experience. If not, code 0. |
| 2 | **Empirical knowledge** Comment based on knowledge gained through scientific evidence (scientific articles or books) |
| 3 | **Second-hand professional knowledge** Comments made by a non-professional that contain information gleaned from a professional source (e.g., a doctor). |
| 4 | **Second-hand unprofessional knowledge** Comments made by a non-professional that contain information gleaned from a non-professional source (e.g., a spiritual healer, shaman). |
| 5 | **Indirect anecdotal evidence** Comments that express knowledge gained from personal experience of someone else, and someone’s participation in a particular phenomenon (e.g., knowledge about how to deal with depression gained by witnessing or hearing about coping with one’s own depression). |
| 6 | **Professional knowledge** |

E.g., to be coded 0 *"(referring to antidepressants) in reality there is always a percentage of non-responders."*

E.g., to be coded 1 "*I have been taking 30mg of Buspar a day for the past four months and after getting over the initial side effects (1-2 months), I found it to be pretty good*."

E.g., to be coded 2 "*I still remember when I read a scientific article where it was said that depression does not become chronic* ..."

E.g., to be coded 3 "*My doctor doesn't think they [several new drugs] are so different that he immediately justifies trying them*"

### V18a,b Misinformation

1. *Misinformation*

| 0 | Advice giving does not contain misinformation |
| --- | --- |
| 1 | Advice giving contains “content misinformation”: a claim that used poor-quality or no clinical evidence in support |
| 2 | Advice giving contains “context misinformation”: the advice-giver has not the status or enough information to warrant accuracy |
| 3 | Wrong assumption (only for advice-seeking): e.g., asking for an diagnosis on an online support group, stating that medications do not work and requesting for alternative therapies |
| 4 | Use of wrong terminology |

E.g., to be coded 1 "*Homeopathy is better than antidepressants"*

E.g., to be coded 2 ”*From the symptoms you describe you certainly suffer from anxiety*”

E.g., to be coded 3 "*Does anyone know how to cure depression without drugs?”*

E.g. to be coded 4 *“Depression is a psychogenic melancholy”*

1. *Misinformation correction*

| 0 | No misinformation correction |
| --- | --- |
| 1 | Misinformation correction |

E.g. to be coded 1”*That’s not true: it is possible to recover from depression!”*

### V19 The illness trajectory and related themes

Here the presence or absence (mentioning or non-mentioning) of parts of the medical process are coded.

| 1 | **Causes and risk factors.** Causes necessarily come in the early stage of the trajectory. As their efficacy has done its part when the disease moves into a patient’s focus, its causes are not as interesting anymore as they would have been, had a patient been aware of what condition s/he would develop. This appears in the trajectory as prevention.  *E.g., Lifestyle, family history, brain chemistry, trauma or stress* |
| --- | --- |
| 12 | **Cause/risk factors in relationship with prevention.** *E.g., a locutor asks advice on how to prevent a depressive episode acknowledging that a member of his/her family is also suffering from depression* |
| 15 | **Causes in relationship with treatment.** *E.g., a locutor asks whether there are differences in treatment options for endogenous or exogenous depression* |
| 2 | **Prevention.** If causes are known or assumed, a rational decision can be made of what could be done, for instance, if it is known or believed that lack of exercise causes coronary disease, you have to exercise more to protect yourself from this condition.  *E.g., Intervening before health effects occur, screening to identify diseases in the earliest*, *getting routine medical care* |
| 3 | **Symptoms.** Receiving information of newly noticed or in fact new symptoms is expected to be a frequent subject on support websites, and a frequent motive for posting an origin locution. Attention to and worry about symptoms will be linkable to diagnosis. The corresponding locutions are statements on which symptoms point to which diseases. A symptom is subjective, that is, apparent only to the patient (for example fatigue or depressed mood), a sign is any objective evidence of a disease that can be observed by others (for example sudden loss of body weight). *E.g., Subjective or objective evidence of disease* |
| 34 | **Symptoms in relationship with diagnosis**. These are all statements that deal with the relationship of symptoms and the correctness of diagnosis. *E.g., A locutor asks if mood swings are a sign of bipolar disorder* |
| 35 | **Symptoms in relationship with treatment.** *E.g., A locutor asks whether antidepressants are a good treatment option given that s/he suffers from depression with atypical feature* |
| 4 | **Diagnosis.** This cornerstone means the diagnosis or condition that is talked about in the utterance, not a diagnosis anyone on the support group might or might not have. Categories of diseases are from the DSM5. So is the structure of the conditions, for that enables coding imprecise use of language**.** *E.g., Differential diagnosis, misdiagnosis, encounter-to-diagnosis lag time, diagnostic criteria, self-diagnosis* |
| 45 | **Diagnosis in relationship with treatment.** *E.g., A locutor asks what the better treatment option is as s/he has recently been diagnosed with seasonal depression.* |
| 5 | **Treatment**. Treatment decisions in reality often have several criteria to consider and weigh against one another. All of the utterance categories are, of course, situation-specific, and the treatment recommended may not be specific and in discussions often be reduced to the question: Psychotherapy or Medication. *E.g., Past medical history, decisions related to treatment options (unrelated to side-effects problems)* |
| 56 | **Treatment in relationship with prognosis.** *E.g., A locutor asks whether cognitive psychotherapy works* |
| 6 | **Prognosis.** Desired and undesired outcomes of treatment options.  *E.g., Disease burden, treatment effectiveness, medication interruption, life expectance, adherence problems* |

# References

1. Introduction to the Special Issue on Language in Social Media: Exploiting Discourse and Other Contextual Information | Computational Linguistics | MIT Press. Accessed February 17, 2022. https://direct.mit.edu/coli/article/44/4/663/1608/Introduction-to-the-Special-Issue-on-Language-in

2. Strijbos JW, Stahl G. Methodological issues in developing a multi-dimensional coding procedure for small group chat communication. *Learn Instr*. 2007;17:394-404.

3. What names are allowed on Facebook? | Facebook Help Centre. Accessed February 17, 2022. https://www.facebook.com/help/112146705538576

4. Taraszow T, Aristodemou E, Shitta G, Laouris Y, Arsoy A. Disclosure of personal and contact information by young people in social networking sites: An analysis using Facebook profiles as an example. *Int J Media Cult Polit*. 2010;6(1):81-101. doi:10.1386/macp.6.1.81/1
