# Supplementary material for: Misinformation in Italian Online Mental Health Communities During the COVID-19 Pandemic: Protocol for a Content Analysis Study
Source: JMIR Res Protoc. 2022 May 20;11(5):e35347. doi: 10.2196/35347 (PMC9166639; doi:10.2196/35347)
Supplement: Multimedia Appendix 2 [file resprot_v11i5e35347_app2.docx]

Multimedia Appendix 2: Reactions and emojis

This is a Multimedia Appendix to a full manuscript published in the J Med Internet Res. For full copyright and citation information see <http://dx.doi.org/10.219/35347>

Table 1. Reactions and Emojis

| **Reaction** | **Reaction’s name** |
| --- | --- |
| **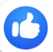** | Like |
| *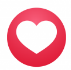* | Love |
| *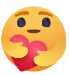* | Care |
| *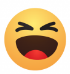* | Haha |
| *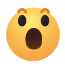* | Wow |
| *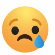* | Sad |
| *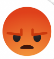* | Angry |

For a complete list on how to code emojis visit the following website: <https://trucchifacebook.com/download/emoji/emoji-commenti-facebook.htm>
