# Supplementary material for: Misinformation in Italian Online Mental Health Communities During the COVID-19 Pandemic: Protocol for a Content Analysis Study
Source: JMIR Res Protoc. 2022 May 20;11(5):e35347. doi: 10.2196/35347 (PMC9166639; doi:10.2196/35347)
Supplement: Multimedia Appendix 3 [file resprot_v11i5e35347_app3.docx]

Multimedia Appendix 3: Summary of variables

This is a Multimedia Appendix to a full manuscript published in the J Med Internet Res. For full copyright and citation information see <http://dx.doi.org/10.219/35347>

**Legenda:**

**⁪** = Variable to code both for advice seeking and giving behaviours
⁪ = Variable to code for advice seeking behaviours
⁪ = Variable to code for advice giving behaviours

***** = possibility to code more than one category

| **Formal variables** | V1 | Bundle number |  |
| --- | --- | --- | --- |
|  | V2 | Sorting variable |  |
|  | V3 | Number of tot. comments |  |
|  | V4 | Locution date | MM. YYYY format |
|  | V5 | Reactions |  |
| **Locutor variables** | V6 | Locutor ID |  |
|  | V7 | Locutor gender | 1 = Female 2 = Male |
|  | V8 | Locutor status | 10 = Locutor is a patient 20 = Locutor is a moderator  Locutor is a caregiver, his/her role is: 31 = Patient’s partner  32 = Patient’s parent 33 = Patient’s daughter /son 34 = Patient’s sister /brother 35 = Patient’s friend, neighbor, acquaintance 36 = Other relative (specify)  40 = Ex patient |
|  | V9 | Medico-scientific qualification of locutor | 0 = no information  1 = approbation as psychologist, psychiatrist, neurologist and comparable disciplines 2 = approbation in other medical fields 3 = other, non-medical but relevant qualification  4 = Alternative healer |
| **Content variables** | V10 | Seeking vs. giving | 0 = Neither giving or seeking is indicated  1 = Seeking  2 = Giving |
|  | V11 | Motivation for “seeking” | NA = not seeking  1 = Declarative knowledge 2 = Procedural knowledge 3 = Decision-making 4 = Emotional support seeking |
|  | V12 | Action through “giving” locution | NA = not giving-behaviour 1 = Declarative knowledge 2 = Procedural knowledge 3 = Call to action  4 = Emotional support |
|  | V13 | Type of Illness ***** | 1 = Depressive and related disorders 2 = Anxiety and related disorders 3 = Bipolar and related disorders 4 = Obsessive-Compulsive and related Disorders 5 = Substance-Related and Addictive Disorders 6 = Personality disorders 7 = Other mental illnesses 8 = Unspecified mental illness 9 = Physical illness 10 = Covid and covid vaccine  11 = Suicidal ideation |
|  | V14 | Treatment options ***** | 1. = No treatment mentioned   1= Psychotherapy in general   1. = Cognitive behavioural therapy 2. = Psychodynamic therapies 3. = Other types of psychotherapy   2 = General medications  21 = Drugs generally used to treat depression 22 = Drugs generally used to treat anxiety  23 = Drugs to treat psychotic disorders   1. = Mood stabilizers 2. = Drugs generally used to treat insomnia 3. = Other types of medication   3 = Mental and mind-body interventions  4 = Alternative physical interventions  5 = Surgical intervention  6 = Hospitalization 7 = Covid vaccine |
|  | V15a | Treatment evaluation | NA = No treatment mentioned  1 = Treatment – neutral 2 = Treatment judged positively  3 = Treatment judged negatively  4 = Mixed |
|  | V15b | Treatment adverse-effects | 0 = No adverse effect mentioned 1 = There is a mention of adverse effects |
|  | V15c | Treatment interruption | 0 = No treatment interruption mentioned 1 = There is mention of treatment interruption |
|  | V16a | Health professional mentioned ***** | 0 = No 1 = Psychologist or psychotherapist 2 = Psychiatrist 3 = Unspecified mental health professional 4 = Other health professional 5 = Non-professional source (e.g., a spiritual healer, shaman) |
|  | V16b | Sentiment towards health professional | NA = Health professional not mentioned  1 = Do not express  2 = Health professional judged positively 3 = Health professional judged negatively 4 = Mixed opinion on health professional |
|  | V16c | Doctor patient relationship | NA Doctor-patient relationship not mentioned  0 = Do not express 1 = Doctor-patient relationship judged positively  2 = Doctor-patient relationship judged negatively |
|  | V17 | Argument quality | NA = not giving-behaviour 0 = No explicit reference to the argument quality  1 = Direct anecdotal evidence 2 = Empirical knowledge 3 = Second-hand professional knowledge 4 = Second-hand unprofessional knowledge  5 = Indirect anecdotal evidence  6 = Professional knowledge |
|  | V18 | Misinformation * | 0 = No misinformation 1= Content misinformation 2 = Context misinformation  3 = Wrong assumption (only for advice-seeking)  4 = Wrong terminology (specifier) |
|  |  | Misinformation correction | 0 = No misinformation correction  1 = Misinformation correction |
|  | V19 | Illness trajectory and themes | NA  1 = Causes and risk factors.  12 = Cause/risk factors in relationship with prevention  15 = Causes in relationship with treatment  2 = Prevention.  3 = Signs and symptoms.  34 = Symptoms in relationship with diagnosis.  35 = Symptoms in relationship with treatment  4 = Diagnosis.  45 = Diagnosis in relationship with treatment  5 = Treatment  56 = Treatment in relationship with prognosis  6 = Prognosis |
| **Assessment variables** | V20 | Correctness | 1 = Coder has a severe level of uncertainty 2 = Coder has a moderate level of uncertainty 3 = Coder is certain on correctness |
