# Supplementary material for: Misinformation in Italian Online Mental Health Communities During the COVID-19 Pandemic: Protocol for a Content Analysis Study
Source: JMIR Res Protoc. 2022 May 20;11(5):e35347. doi: 10.2196/35347 (PMC9166639; doi:10.2196/35347)
Supplement: Multimedia Appendix 4 [file resprot_v11i5e35347_app4.docx]

Multimedia Appendix 4: Medication types

This is a Multimedia Appendix to a full manuscript published in the J Med Internet Res. For full copyright and citation information see <http://dx.doi.org/10.219/35347>

Source: <https://www.nimh.nih.gov/health/topics/mental-health-medications>. The list is in the Italian language and comprehends active ingredients and drugs.

# Antidepressants

Antidepressants are medications commonly used to treat depression. The most popular types of antidepressants are called selective serotonin reuptake inhibitors (SSRIs). Other types of antidepressants are serotonin and norepinephrine reuptake inhibitors (SNRIs).

Azur

Bupropione

Cipralex

Citalopram

Clexiclor

Cloriflox

Dapagut

Daparox

Diesan

Dropaxin

Dumirox

Elopram

Entact

Escitalopram

Eutimil

Felipram

Feliximir

Fevarin

Flotina

Fluoxeren

Fluoxetina

Fluvoxamina

Frimaind

Frimaind

Ipsumor

Kaidor

Marpram

Maveral

Paroxetina

Percitale

Prozac

Return

Ricap

Sereupin

Seropram

Seroxat

Sertralina

Sintopram

Stiliden

Tatig

Tralisen

Verisan

Xeredien

Zoloft

# Anti-anxiety

Anti-anxiety medications help reduce the symptoms of anxiety, such as panic attacks, or extreme fear and worry.

Benzodiazepine

Clonazepam

Alprazolam

Lorazepam

Tavor

Rivotril

Xanax

Valium

Ansiolin

En

Frontal

Lexotan

Prazene

Control

Lorans

Dalmadorm

Felison

Halcion

Minias

Roipnol

Nottem

Stilnox

Buspar

# Antipsychotics

Antipsychotic medicines are primarily used to manage psychosis.

Acetofenzaina

Aloperidolo

Clorpromazina

[Clozapina](https://www.my-personaltrainer.it/salute-benessere/clozapina.html)

Droperidolo

Flufenazina

Loxapina

[Olanzapina](https://www.my-personaltrainer.it/salute-benessere/olanzapina.html)

[Perfenazina](https://www.my-personaltrainer.it/salute-benessere/perfenazina.html)

Perfenazina

[Proclorperazina](https://www.my-personaltrainer.it/benessere/proclorperazina.html)

[Quetiapina](https://www.my-personaltrainer.it/salute-benessere/quetiapina.html)

Spiperone

[Sulpiride](https://www.my-personaltrainer.it/salute-benessere/sulpiride.html)

Tioridazina

Trifluperido

# Mood stabilizers

Mood stabilizers are used primarily to treat bipolar disorder, mood swings associated with other mental disorders, and in some cases, to augment the effect of other medications used to treat depression.

Litio

Valproato di Sodio

Carbamazepina

Lamotrigina

Oxcarbamazepina
